# Addressing Emotional Wellness During the COVID-19 Pandemic: the Role of *Promotores* in Delivering Integrated Mental Health Care and Social Services

**DOI:** 10.5888/pcd18.200656

**Published:** 2021-05-27

**Authors:** Kyle J. Moon, Gloria Itzel Montiel, Patricia J. Cantero, Saira Nawaz

**Affiliations:** 1 Center for Health Outcomes and Policy Evaluation Studies, Ohio State University College of Public Health, Columbus, Ohio; 2 Latino Health Access, Santa Ana, California

## Abstract

**Introduction:**

The disproportionate impact of the COVID-19 pandemic on Latino communities has resulted in greater reports of depression, anxiety, and stress. We present a community-led intervention in Latino communities that integrated social services in mental health service delivery for an equity-based response.

**Methods:**

We used tracking sheets to identify 1,436 unique participants (aged 5–86) enrolled in Latino Health Access’s Emotional Wellness program, of whom 346 enrolled in the pre–COVID-19 period (March 2019–February 2020) and 1,090 in the COVID-19 period (March–June 2020). Demographic characteristics and types of services were aggregated to assess monthly trends using Pearson 𝜒^2^ tests. Regression models were developed to compare factors associated with referrals in the pre–COVID-19 and COVID-19 periods.

**Results:**

During the pandemic, service volume (*P* < .001) and participant volume (*P* < .001) increased significantly compared with the prepandemic period. Participant characteristics were similar during both periods, the only differences being age distribution, expanded geographic range, and increased male participation during the pandemic. Nonreferred services, such as peer support, increased during the pandemic period. Type of referrals significantly changed from primarily mental health services and disease management in the prepandemic period to affordable housing support, food assistance, and supplemental income.

**Conclusion:**

An effective mental health program in response to the pandemic must incorporate direct mental health services and address social needs that exacerbate mental health risk for Latino communities. This study presents a model of how to integrate both factors by leveraging *promotor*-led programs.

**Summary**

**What is already known on the topic?**

Mental health needs have been exacerbated by the COVID-19 pandemic. As a result, Latino communities experience disparate rates of stress, depression, and anxiety.

**What is addressed by this report?**

Few studies explore *promotor*-led mental health interventions as strategies to address service gaps in Latino communities. This article describes a community-based intervention that integrates social services and mental health services.

**What are the implications for public health practice?**

With ongoing COVID-19 surges and with vaccine distribution underway, a critical need remains to respond with equity. Latino Health Access’s Emotional Wellness program emphasizes the importance of delivering mental health care integrated with social services and provides a model to reduce the effect of COVID-19 in socioeconomically disadvantaged communities.

## Introduction

Mental health needs of working-class Black and Latino communities have long been insufficiently met in the United States (1). The COVID-19 pandemic has exacerbated mental health needs through unpredictability and uncertainty, physical distancing, social isolation, loss of employment and income, mortality, and social suffering (2). Among US adults surveyed in June 2020, 52.1% of Hispanic adults reported at least 1 adverse mental or behavioral health condition, compared with 37.8% of non-Hispanic White adults. Hispanic adults reported higher prevalence of anxiety or depressive disorder, trauma-related and stressor-related disorder, substance use to cope with stress, and suicidal ideation (3). These disparities in mental health effects reflect the grief, bereavement, and stress related to financial insecurity resulting from the pandemic in Latino communities (4), which along with other racial and ethnic minority communities, have been disproportionately affected by COVID-19 as a result of structural racism (5).

Experts have called for local implementation (6) of community-level mental health interventions and prevention efforts that integrate financial relief and social services, promote social cohesion, and provide culturally and linguistically tailored education on COVID-19 and mental health (2,3). The American Psychological Association has also called for a “reimagining” of the behavioral health system as one that reaches people where they are, recognizes wisdom in each community to solve its own problems, and looks to innovative roles for new mental health practitioners who are firmly rooted in their communities (6,7). Responding to these calls to action, this study investigated the role of *promotores de salud* (community health workers) in providing community-led and integrated mental health care and social services in response to the COVID-19 pandemic in Latino communities of Orange County, California. Our findings may provide a model for integrating equity in mental health interventions during and after the pandemic.

## Methods

### Emotional Wellness program framework

Latino Health Access’s (LHA’s) *Bienestar Emocional* (Emotional Wellness) program draws on principles of narrative therapy and aligns with human-centered design, which prioritizes participant engagement throughout the lifecycle of the program (8,9). The program was developed by LHA *promotores* in partnership with a marriage and family therapist trained in narrative therapy. Narrative therapy recognizes participants as authors of their own stories, in which we are all participants in each other’s stories (10) and empowers people to write a new story as a process to overcome the inequities and oppressions of the dominant social narrative (11). In this way, narrative therapy can link people with similar stories, joining their voices together in shared purpose to improve their mental and emotional well-being (10). Narrative therapy has demonstrated success in overcoming stigma associated with therapy and social position because it centers the person rather than imposing a hierarchy, with the counselor as expert (11). A unique feature of LHA’s program is that it is facilitated by *promotores* with ongoing training and support provided by a marriage and family therapist.

**Figure 1:**
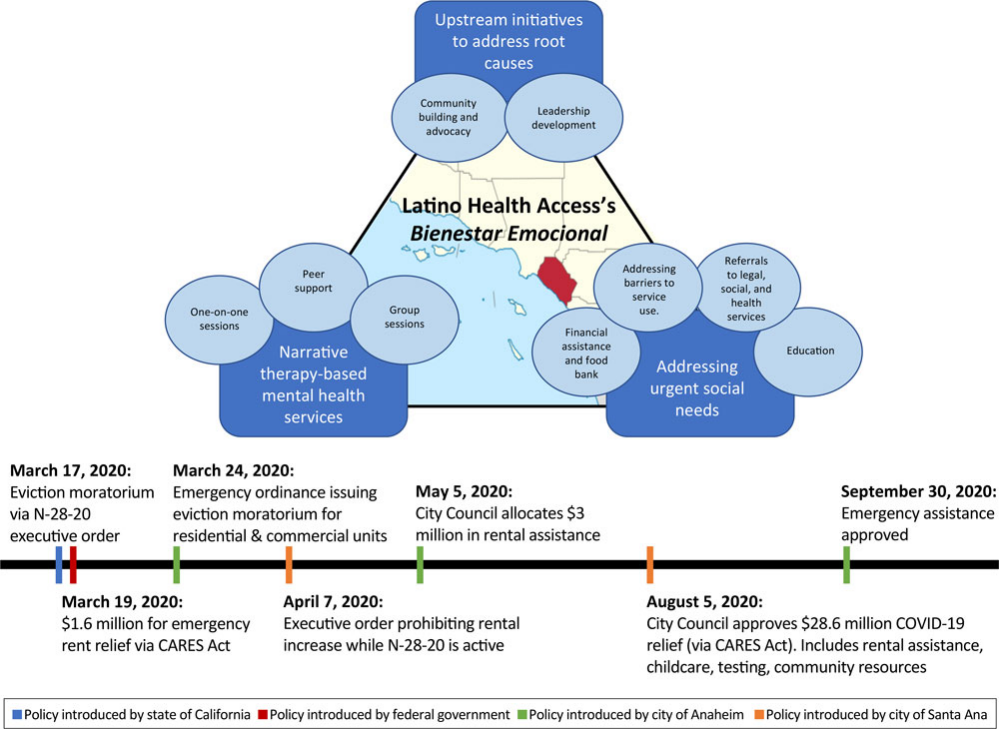
Framework for Latino Health Access’s *Bienestar Emocional* (Emotional Wellness) program describing its 3 primary initiatives, their components, and the associated timeline of related events. The program is based in Orange County, California. Abbreviations: CARES, Coronavirus Aid, Relief, and Economic Security Act; N-20-28, executive order issued by Governor Newsom of California that allows local governments to impose temporary limitations on residential and commercial evictions under COVID-19-related financial distress.

The multipronged nature of the Emotional Wellness program addresses the spectrum of needs for the community, such that 1) narrative therapy and peer support achieve culturally appropriate mental health services, 2) services to overcome barriers to care address more immediate health and social needs, and 3) community advocacy and leadership are intended to address inequities by shifting the policy environment (Figure 1). Because of the its reach and grounding in human-centered design, along with the trusted relationships *promotores* have with participants as program facilitators and community members facing similar circumstances, the program was the appropriate vehicle for providing integrated care once COVID-19 hit communities in early March 2020. The Emotional Wellness program was enhanced to expand delivery of mental health services while addressing social needs of food and housing insecurity. Through these services, LHA ensured its mental health response was rooted in addressing the social inequities that created the conditions by which COVID-19 devastated working-class racial and ethnic minority communities and exacerbated mental health stressors (12). The Emotional Wellness program adopted a population health approach to address behavioral health needs along a continuum, regardless of whether participants had a mental or emotional health condition, providing a range of services (6). As COVID-19 policies took effect (Figure 1), the Emotional Wellness program was well-positioned to expand to help vulnerable groups meet their immediate mental health and social needs, while continuing to address the structural inequities exacerbated by the pandemic.

### Study design

Our observational study used de-identified tracking data collected by LHA over 2 years (March 2019–June 2020). No sampling was conducted, because the full universe of clients was needed to reflect changes in volume of services received (mental health and social services) and referrals provided during the prepandemic and pandemic periods. Because data were stripped of all identifiable information with no linkage to the participants from whom it was originally collected, the study did not constitute human subjects research and therefore did not require internal review board approval.

### Study site and participants

LHA, a nonprofit public health organization in Santa Ana, California, partners with Latinx communities in Orange County to advance health equity through a combination of culturally and linguistically concordant direct services and upstream initiatives that address social determinants of health through community-led policy, systems, and environmental change. Programs are facilitated by *promotores*, who are members of the community and thus, have a wealth of local knowledge and expertise, understand the lived experiences of those in the community, and have specialized training in health promotion and community advocacy.

All participants in this study were drawn from LHA’s Emotional Wellness program from March–June 2019 and January–June 2020, during which 1,436 unique participants were enrolled. All participants were recruited from Orange County, California, with participants representing 25 of the 34 cities and 50 of the 88 zip codes in the county. Historic data showed that LHA participants were predominantly female (72%), aged 18 or older (71%), Latino (98%), uninsured (46%), foreign born (95%), Santa Ana residents (78%), monolingual Spanish speakers (90%), and earned less than $30,000 annually (85%) (13).

### Procedure

*Promotores* across all LHA programs referred participants to the *Bienestar Emocional* program whom they identified as having experienced or were currently experiencing trauma or domestic violence. Once enrolled, emotional wellness *promotores* conducted an exploratory session to uncover the priority issues for the participant and identify their most pressing social needs. Thereafter, participants engaged in group sessions and one-on-one sessions with a *promotor* by using a curriculum based on principles of narrative therapy. During each session, *promotores* provided a range of interventions, including education (navigating legal, medical, education, penal, or immigration systems), peer support (donations, goal setting, identifying strengths and barriers, moral support, system support), leadership development (advocacy and individual coaching), community building and engagement (group projects, activities, volunteering), addressing barriers to service use (application assistance, childcare, health care access, translation services, transportation arrangements), and referrals to legal, social, and health services. All *promotores* were trained by a marriage and family therapist to facilitate structured sessions through the program curriculum. In addition, *promotores* received training on mental health and community interventions from a bilingual and bicultural therapist.

Once COVID-19 struck, service delivery changed: group sessions convened via video conferencing, and one-on-one sessions were carried out over the telephone. In March 2020, LHA rapidly expanded its referrals to address social needs, with COVID-19 financial relief, nutrition assistance, and affordable housing support. As such, participant volume also increased substantially during the pandemic, because 1) social needs proliferated and 2) *promotores* organized an initial COVID-19 pandemic response by calling current and prior LHA participants to understand their experiences with the pandemic in real time. During these calls, *promotores* provided prevention information, education, and resources as well as presented civic engagement opportunities to address the rising housing crisis, the decennial census, and the 2020 Presidential election with nonpartisan voter engagement messaging.

### Data collection and statistical analysis

During one-on-one sessions, data were captured by each *promotor* by using a 12-character unique identifier. Service providers removed all personal information and shared the de-identified data with Ohio State University researchers (K.J.M. and S.N.). Analyses were conducted to compare the effects of the pandemic on provision of services. First, demographic characteristics were compared for the sample of participants in the prepandemic (March 2019–February 2020) and the pandemic (March 2020–June 2020) periods. Demographics included age, ethnicity, sex, and geographic residence.

Second, analyses of services used were conducted at 2 levels, 1) by service volume and type, and 2) by participant. For analyses by participant, service use trends were controlled such that each participant received a maximum of 1 of each service during a particular month. For example, if Participant A had 3 service encounters for peer support and 1 service encounter for education during March, Participant A received 2 services during March: peer support and education. The denominator for participant-based analyses was the number of unique participants in each month. We used independent *t* tests to assess differences in service and participant volume from the prepandemic to pandemic period. The Pearson 𝜒^2^ test of independence was used to identify significant differences in demographic characteristics and service use by service type and referral category. Yates’s continuity correction was applied when any cell in the contingency table had a frequency less than 10. Significance was established at α = .05; 95% CIs were constructed for all proportions. To assess variation across the ten months, an overall *P* value was computed, and a second comparison was computed between prepandemic (March 2019–February 2020) and pandemic (March 2020–June 2020) periods. The third analysis involved the development of logistic regression models to compare predictors of participants receiving referrals in the prepandemic and pandemic periods. Participants with missing data for 1 or more demographic variable(s) were excluded from the regression models, as were participants enrolled during both prepandemic and pandemic periods. We included 722 unique participants in the analysis, of whom 210 were enrolled during the prepandemic period and the remaining 512 during the pandemic period. All analyses were performed by using R Statistical Software, version 3.6.2 (R Foundation for Statistical Computing).

## Results

We enrolled 1,436 unique participants in LHA’s Emotional Wellness program from March 2019 to June 2020. Of these, 660 participants (46.0%) were excluded from demographic analyses because of missing data, leaving 776 unique participants, 57 of whom were enrolled in the Emotional Wellness program during both the prepandemic and pandemic periods. The magnitude of missing data is largely due to the transition to virtual service delivery and the rapid expansion of the program in response to COVID-19. Of the 776 unique participants, most were Latino (n = 763, 98.3%), female (n = 594, 76.5%), aged 25–44 (n = 400, 51.5%), and from Santa Ana (n = 503, 64.8%) (Table 1). Group differences in sex (increased male participation during the pandemic period, *P* < .001), age (decreased participation among people aged 5–17 [12.8% vs 4.1%, *P* < .001] and increased participation among people aged 45–64 [30.1% vs 37.9%, *P* = .03] during the pandemic period), and geographic residence (decreased participation from Anaheim [20.3% vs 14.3%, *P* = .03] and increased participation from other cities [12.0% vs 22.2%, *P* < .001]) were significant. Groups did not differ by ethnicity (98.1% Latino prepandemic vs 98.6% pandemic, *P* = .83).

**Table 1:** Demographic Characteristics of Participants (N = 722) in Latino Health Access’s Emotional Wellness Program During the COVID-19 Pandemic, Orange County, California^a^

Characteristic	Pre-COVID-19 (March 2019-February 2020), n = 266^b^	COVID-19 (March 2020-June 2020), n = 567^b^	*P* Value^c^
**Ethnicity**			
Hispanic/Latino	261 (98.1) [96.5–99.8]	559 (98.6) [97.6–99.6]	.83
Other	261 (1.9) [0.2–3.5]	8 (1.4) [0.4–2.4]	
**Sex**			
Male	40 (15.0) [10.7–19.3]	146 (25.7) [22.2–29.3]	<.001
Female	226 (85.0) [80.7–89.3]	421 (74.3) [70.7–77.8]	
**Age, y**			
5–17	34 (12.8) [8.8–16.8]	23 (4.1) [2.4–5.7)	<.001
18–24	6 (2.3) [0.5–4.0]	9 (1.6) [0.6–2.6]	.69
25–44	133 (50.0) [44.0–56.0]	298 (52.6) [48.4–56.7]	.49
45–64	80 (30.1) [24.6–35.6]	214 (37.9) [33.9–41.9]	.03
≥65	13 (4.9) [2.3–7.5]	22 (3.9) [2.3–5.5]	.50
**Location of residence**			
Santa Ana	180 (67.7) [62.0–73.3]	360 (63.5) [59.5–67.5]	.24
Anaheim	54 (20.3) [15.5–25.1]	81 (14.3) [11.4–17.2]	.03
Other	32 (12.0) [8.1–15.9]	126 (22.2) [18.8–25.6]	<.001

a Fifty-seven participants were enrolled in both prepandemic and pandemic programs.

b Values are number (percentage) [95% CI] unless otherwise indicated.

c Pearson 𝜒^2^ test of independence was used to determine significance, with Yates’ continuity correction applied when any cell had a frequency of <10. *P* value <.05 considered significant. *P* value assesses difference between prepandemic (March 2019-February 2020) and pandemic period (March 2020-June 2020).

### Trends in use of services

**Figure 2:**
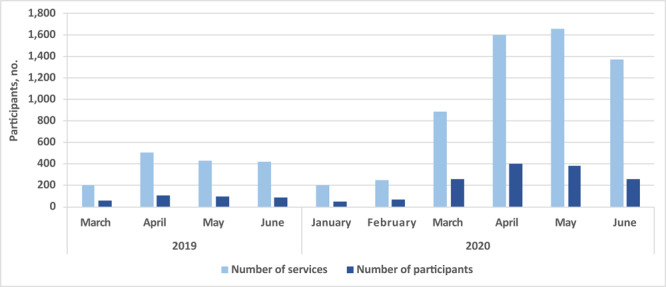
Service use among Latino Health Access’s Emotional Wellness participants, showing trends in volume of services and participants during 10 months (March 2019–June 2020). The ratio of services to participants increased from an average of 4.0 in the pre-COVID-19 period to an average of 4.3 in the COVID-19 period (*P* = .54). Significance was assessed by using an independent *t* test.

From prepandemic to pandemic periods, the volume of services (*P* < .001) and participants (*P* < .001) increased significantly (Figure 2). Although the volume of participants was driven, in part, by the *promotores*’ COVID-19 outreach, the ratio of services to participants increased, though not significantly, from an average of 4.0 in the prepandemic period to 4.3 in the pandemic period (*P* = .54), meaning each participant received a greater number of services.

**Table:** 

Service Use	2019				2020						Prepandemic vs Pandemic, *P* Value
	March	April	May	June	January	February	March	April	May	June	
Volume of services	201	508	434	419	200	251	890	1,596	1,652	1,375	.54
Volume of participants	59	106	101	93	56	75	260	400	381	256	

Trends in referrals varied significantly in 8 of the 9 service categories in both periods (Table 2).When the COVID-19 pandemic struck in full force in March 2020, a significant uptick in referrals occurred for affordable housing (*P* < .001), financial assistance (*P* < .001), and food and nutrition assistance (*P* < .001). Paradoxically, referrals to mental health services declined steeply (*P* < .001) as did referrals for health education and disease management (*P* < .001) from the prepandemic to pandemic period.

**Table 2:** Trends in Referrals to Mental Health and Social Services Among Participants (N = 722) in Latino Health Access’s Emotional Wellness Program During the COVID-19 Pandemic, Orange County, California^a^

Category	2019				2020						Overall *P* Value^b^	Pre-COVID-19 vs COVID-19 *P* Value
	Mar, n = 45	Apr, n = 69	May, n = 75	Jun, n = 66	Jan, n = 44	Feb, n = 67	Mar, n = 220	Apr, n = 307	May, n = 344	Jun, n = 224		
Affordable housing	0	4.3 (0–9.2)	0	3.0 (0–7.2)	2.3 (0–6.7)	1.5 (0–4.4)	13.6 (9.1–18.2)	13.0 (9.3–16.8)	10.5 (7.2–13.7)	12.1 (7.8–16.3)	<.001	<.001
Financial assistance	0	0	0	0	0	0	0.9 (0–2.2)	30.9 (25.8–36.1)	61.0 (55.9–66.2)	55.4 (48.8–61.9)	<.001	<.001
Food and nutrition assistance	0	1.4 (0–4.3)	0	1.5 (0–4.5)	0	0	74.1 (68.3–79.9)	61.6 (56.1–67.0)	45.9 (40.7–51.2)	49.6 (43.0–56.1)	<.001	<.001
Health education and disease management	62.2 (48.1–76.4)	34.8 (23.5–46.0)	36.0 (25.1–46.9)	7.6 (1.2–14.0)	15.9 (5.1–26.7)	4.5 (0–9.4)	0	2.0 (0.4–3.5)	4.4 (2.2–6.5)	7.1 (3.8–10.5)	<.001	<.001
Legal services and advocacy	4.4 (0–10.5)	5.8 (0.3–11.3)	9.3 (2.7–15.9)	12.1 (4.2–20.0)	9.1 (0.6–17.6)	9.0 (2.1–15.8)	2.3 (0.3–4.2)	3.9 (1.7–6.1)	3.5 (1.5–5.4)	6.3 (3.1–9.4)	.02	<.001
Medical care	6.7 (0–14.0)	1.4 (0–4.3)	2.7 (0–6.3)	4.5 (0–9.6)	11.4 (2.0–20.7)	4.5 (0–9.4)	2.3 (0.3–4.2)	4.2 (2.0–6.5)	9.0 (6.0–12.0)	25.9 (20.2–31.6)	<001	.002
Mental health services	4.4 (0–10.5)	33.3 (22.2–44.5)	28.0 (17.8–38.2)	45.5 (33.4–57.5)	72.7 (59.6–85.9)	83.6 (74.7–92.5)	12.3 (7.9–16.6)	6.8 (4.0–9.7)	2.3 (0.7–3.9)	6.7 (3.4–10.0)	<.001	<.001
Recreation	88.9 (79.7–98.1)	34.8 (23.5–46.0)	48.0 (36.7–59.3)	40.9 (29.0–52.8)	2.3 (0–6.7)	1.5 (0–4.4)	0.5 (0–1.3)	0.3 (0–1.0)	0	0	<.001	<.001
Other	2.2 (0–6.5)	7.2 (1.1–13.4)	2.7 (0–6.3)	4.5 (0–9.6)	0	3.0 (0–7.1)	0.9 (0–2.2)	2.9 (1.0–4.8)	5.5 (3.1–7.9)	3.1 (0.8–5.4)	.18	.93

a Values are percentage (95% CI) unless otherwise indicated.

b Pearson 𝜒^2^ test of independence was used to determine significance, with Yates’ continuity correction applied when any cell had a frequency of <10. *P* value <.05 considered significant.

### Regression models

Results from logistic regression analysis identified factors associated with the receipt of referrals (Model 1), receipt of referrals for mental health services (Model 2), and receipt of referrals to address social needs (Model 3) (Table 3). During the prepandemic period, 179 (85.2%) received 1 or more referrals; 104 (58.1%) received a referral for mental health services. In March 2020, 475 (92.8%) received 1 or more referrals, of which 20 (4.2%) were for mental health services, and 416 (87.6%) to address social needs. Of those receiving a referral for mental health services, 13 (65.0%) received referrals to address both mental health and social needs.

**Table 3:** Regression Analysis of Likelihood of Referral to Mental Health or Social Services Among Participants (N = 722) in Latino Health Access’s Emotional Wellness Program During the COVID-19 Pandemic, Orange County, California^a^

Predictors	Model 1: ≥1 Referral		Model 2: Referred to Mental Health Services		Model 3: Referred to Social Services
	Pre-COVID-19	COVID-19	Pre-COVID-19	COVID-19	
**Sex**					
Female	1 [Reference]	1 [Reference]	1 [Reference]	1 [Reference]	1 [Reference]
Male	−0.41 (−1.70 to 0.89)	0.06 (−0.67 to 0.85)	−0.23 (−0.92 to 0.60)	−0.64 (−1.76 to 0.27)	0.39 (−0.16 to 0.98)
**Age, y**					
<65	1 [Reference]	1 [Reference]	1 [Reference]	1 [Reference]	1 [Reference]
≥65	−0.98 (−3.18 to 1.02)	17.04 (NA)	−0.27 (−1.88 to 1.19)	−14.78 (−268.94 to 28.79)	−0.06 (−1.21 to 1.43)
**Location of residence**					
Santa Ana	1 [Reference]	1 [Reference]	1 [Reference]	1 [Reference]	1 [Reference]
Outside Santa Ana	0.70 (−0.33 to 1.80)	−0.64 (−1.35 to 0.05)	−1.06^b^ (−1.66 to (−0.41)	−1.59^b^ (−2.83 to (−0.61)	0.21 (−0.29 to 0.73)
**Number of Latino Health Access services**					
None	1 [Reference]	1 [Reference]	1 [Reference]	1 [Reference]	1 [Reference]
1–3	−20.95 (NA)	−17.46 (−262.92 to 21.14)	−1.91^c^ (−2.97 to (−1.00)	−1.17^d^ (−2.22 to (−0.24)	−0.19 (−0.75 to 0.36)
4 or 5	−19.56 (−399.72 to 59.69)	−17.32 (−262.78 to 21.28)	−0.40 (−1.12 to 0.31)	0.28 (−0.58 to 1.11)	−0.17 (−0.81 to 0.48)

Abbreviation: NA, not available.

a Values are odds ratio (95% CI). All models were developed as logistic regressions. Model 3, Social Services, was implemented in March 2020. Wald χ^2^ test was used to determine significance.

b Significant at *P* < .01.

c Significant at *P* < .001.

d Significant at *P* < .05.

During both prepandemic and pandemic periods, participants who resided outside of Santa Ana were significantly less likely to receive a referral (odds ratio [OR] = −1.06 during prepandemic, *P* = .001 vs −1.59 pandemic, *P* = .004). During the prepandemic period, participants receiving 1 to 3 services from LHA were significantly less likely than those not receiving services from LHA to receive a referral for mental health services (OR = −1.91, *P* < .001). Although still significant, the OR declined during the pandemic period (−1.17, *P* = .02), meaning the likelihood of participants not receiving mental health services decreased.

## Discussion

Our study aimed to understand the ways in which *promotores* incorporated equity in a COVID-19 community mental health intervention in the Latino communities of Orange County, California. An equity response prioritizes the populations that are most affected by health disparities and engages them in developing strategies to address both the immediate needs and root causes of these disparities. Our intervention leveraged principles of narrative therapy, integrated social services that addressed needs created by structural inequities, and engaged participants in upstream initiatives to address not only gaps in services but the conditions that underlie these gaps. Although prior mental health initiatives sought to improve cultural competency of interventions (7,14), the intervention presented herein was unique in that it incorporated social services as a strategy to build equity in the delivery of community-driven emotional wellness services (15). This integration drew on human-centered design by addressing the community’s social realities directly and centering the experiences of the communities it intended to affect.

Data from our study provide evidence of the association between social needs and mental health needs, because the pandemic period marked a rapid increase in the receipt of in-house mental health and social services. Increases in the volume of services and participants were likely the result of social and economic precarity: reduced work hours and unemployment (and thus, loss of income, food insecurity, and housing instability) during the shelter-in-place period and subsequent business closures. However, mental health and disease management referrals sharply declined as social service referrals increased. Before the pandemic, LHA provided programming in diabetes self-management, obesity prevention, and chronic pain management (16), all of which were *promotor*-led with group and individual meetings. Like mental health needs, disease management needs did not disappear during the pandemic, but because of the economic impact of COVID-19 on Latino communities, it became pressing to provide the referrals and additional services linked to the entrenched social determinants of health that resulted in greater social needs for vulnerable populations (12,17). Therefore, an equitable response to mental health during the pandemic had to, at a minimum, also account for the social needs and heightened stress that affected these communities. An approach that only focuses on adapting mental health interventions for Latino communities may fail without a more integrated approach to care that accounts for social needs. Furthermore, data from our study also suggests that housing, financial assistance, and food are among the most important social needs in the rise of the pandemic, and all 3 of these have been associated with mental health and stability (4,18). In many cases, financial strain — and the resulting poverty, increased exposure to violence, food insecurity, and reduced access to social safety nets — is the fundamental cause of mental health issues (18–20).

LHA Emotional Wellness participants were predominantly from immigrant backgrounds, and a high proportion were uninsured, which may have contributed directly to their reliance on community-based organizations such as LHA for critical mental health and social services for which they may have been ineligible through mainstream systems. At the same time, it may also have been these characteristics that drove a disproportionate need among these participants, in comparison to nonimmigrant Latino participants or those insured either through private insurance or Medicaid. Nonetheless, because the *promotor* model relies on a workforce with local knowledge and expertise, the model can be generalizable to other communities and has already been tested as a model for health education, health promotion, and programmatic interventions to address health outcomes in other ethnic communities in the United States and globally (21,22). Our study advances empirical knowledge on *promotor-*facilitated mental health programming by 1) providing understanding of community-based mental health interventions during the COVID-19 pandemic and 2) describing how leveraging a model built on strong community trust can be an effective vehicle in providing integrated care for mental health and social services. Because traditional health systems have proven less than effective in addressing the community spread of COVID-19 (23), our study showed how *promotor*-led interventions could rapidly address inequities arising from COVID-19 and associated policies, meet social needs, and reduce social isolation, all while mobilizing the community to advocate against racist policies related to housing, employment, and access to social services. LHA’s Emotional Wellness model illustrates how long-term engagement with a community is needed to effectively apply principles of human-centered design in health and social service delivery models to advance equity. As the role of social determinants of health in creating inequalities has become clear during the COVID-19 pandemic (24), many health systems have sought solutions, such as referral systems (25) or payment models (26), to screen patients and link them to services in the community. Though these have been effective in increasing referral rates, the acceptability of these services and their health effects has not been well documented. Because LHA is present in the community, it has helped shape programs proposed by health systems and public health and academic centers, establishing its unique value as part of the COVID-19 response.

Our study has several limitations. We relied on participant tracking data that were collected virtually during the pandemic period, resulting in missing data for demographic characteristics. A comparison of available data (services used, city, zip code) for participants with missing data versus those included in the study produced no significant differences, and we therefore believe our results are generalizable to all LHA participants. The outcome for our study was limited to use of services, and in the absence of a comparison group, we could not establish the effectiveness of the Emotional Wellness program on health outcomes. Previous *promotor-*led interventions in mental health services faced similar challenges, with favorable observations from ethnographic evaluation but no significant improvements in health outcomes (7), underscoring the need for further research to link interventions to mental health outcomes. Additionally, we had no tracking data to determine how many participants who received a referral accessed services at the referred agency. Given LHA’s long-standing presence in Orange County, the organization has forged strong community partnerships for referrals. Where possible, *promotores* established an initial call to a service provider and helped program participants make the initial contact or appointment. *Promotores* also gave participants eligibility information for each service and all contact information for partner agencies. Future interventions are necessary to identify which social needs should be addressed to improve mental health (27). Given LHA’s limited resources, the authors had to rely on existing data to assess program value and identify opportunities for improvement, adaptation, and expansion as the community’s needs evolved. We, however, believe that the study’s benefits outweigh its limitations as the US seeks effective models for addressing ongoing surges in the COVID-19 pandemic and ensuring equitable roll-out of vaccines to reach systematically disadvantaged populations (28,29). Effective communication strategies, with peer-to-peer vaccine education and outreach, may be an effective strategy to address vaccine mistrust and misinformation in Latino communities. Such efforts are likely to ease uncertainty and alleviate stress, and thus, may help address mental health conditions associated with COVID-19 (2,3).

Our study showed how a community-based organization with long-standing ties in the Latino community effectively expanded its Emotional Wellness program to provide integrated mental health care and social services to clients disproportionately affected by the COVID-19 pandemic. Despite some limitations, the study findings are informative for traditional health systems that have struggled to address the health inequities that have been exacerbated during the pandemic. Although social needs have taken precedence, evidence of the mental health toll of the pandemic are already well documented (3), and programs such as LHA’s Emotional Wellness program are needed to reduce the pandemic’s impact in systematically disadvantaged communities.
